# The effect of a partially hydrolysed formula based on rice protein in the treatment of infants with cow’s milk protein allergy

**DOI:** 10.1111/j.1399-3038.2010.00991.x

**Published:** 2010-06

**Authors:** M Reche, C Pascual, A Fiandor, I Polanco, M Rivero-Urgell, R Chifre, S Johnston, M Martín-Esteban

**Affiliations:** 1 Allergy Department, University Children’s Hospital La PazMadrid, Spain; 2 Gastroenterology Department, University Children’s Hospital La PazMadrid, Spain; 3 Research and Development Department, Ordesa Group – Scientific ParkBarcelona, Spain

**Keywords:** cow’s milk protein allergy, hydrolysed rice protein formula, extensively hydrolysed cow’s milk protein formula

## Abstract

Reche M, Pascual C, Fiandor A, Polanco I, Rivero-Urgell M, Chifre R, Johnston S, Martín-Esteban M. The effect of a partially hydrolysed formula based on rice protein in the treatment of infants with cow’s milk protein allergy. Pediatr Allergy Immunol 2010: 21: 577–585. © 2010 John Wiley & Sons A/S

Infants diagnosed with allergy to cow’s milk protein (CMP) are fed extensively hydrolysed cow’s milk formulas, modified soy formulas or even amino acid-based formulas. Hydrolysed rice protein infant formulas have become available and have been shown to be well tolerated by these infants. A prospective open, randomized clinical study to compare the clinical tolerance of a new hydrolysed rice protein formula (HRPF) with an extensively hydrolysed CMP formula (EHF) in the feeding of infants with IgE-mediated cow’s milk allergy. Ninety-two infants (46 boys and 46 girls, mean age 4.3 months, range 1.1–10.1 months) diagnosed with IgE-mediated cow’s milk allergy were enrolled in the study. Clinical tolerance to the formula products was tested. Clinical evaluation included skin prick tests with whole cow’s milk, soya and rice as well as antigens of CMP (beta-lactoglobulin, alpha-lactalbumin, casein and bovine seroalbumin), HRPF and EHF and specific IgE determinations to CMP using CAP technology. Patients were randomized to receive either an EHF based on CMP or a new HRPF. Follow-up was at 3, 6, 12, 18 and 24 months. Growth parameters were measured at each visit. One infant showed immediate allergic reaction to EHF, but no reaction was shown by any infant in the HRPF group. The number of infants who did not become tolerant to CMP during the study was not statistically different between the two groups. Measurement of IgE levels of infants allergic to CMP during the study showed no significant differences between the two formula groups. Growth parameters were in the normal range and similar between groups. In this study, the HRPF was well tolerated by infants with moderate to severe symptoms of IgE-mediated CMP allergy. Children receiving this formula showed similar growth and development of clinical tolerance to those receiving an EHF. In accordance with current guidelines, this HRPF was tolerated by more than 90% of children with CMP allergy and therefore could provide an adequate and safe alternative to CMP-hydrolysed formulas for these infants**.**

Guidelines for the dietary management of infants diagnosed with allergy to cow’s milk protein (CMP) recommend substitution of the cow’s milk formula by extensively hydrolysed formulas based on CMP, modified soy protein formulas and, in certain cases, amino acid-based formulas. Several guidelines have been established for the treatment of CMP allergy as well as indications of the nutritional composition of these infant formulas (1–7).

However, the infant formulas traditionally used in CMP allergy are not without certain difficulties (8–11). A study carried out in infants diagnosed with allergy to CMP showed adverse reactions with a double-blind placebo-controlled food challenge (DBPCFC) to extensively hydrolysed formula (2.2%) and to soy (10%) (12).

Generally, extensively hydrolysed cow’s milk formulas have a bitter taste, and this poor palatability can be a cause of refusal by infants.

Recent publications have recommended that soy protein formula should not be used during the first 6 months of life for infants with food allergy (13, 14). These recommendations therefore limit the use of soy formulas.

As a result, attention has been given to providing further options for feeding infants allergic to CMP, in particular the use of hydrolysed formulas based on rice protein supplemented with L-lysine and L-threonine to achieve an amino acid profile similar to that of breast milk (5, 15).

Studies on hydrolysed rice protein formulas (HRPF) have focused on their possible use for feeding infants allergic to CMP. Two studies by Fiocchi et al. (16, 17) have shown that infants with allergy to CMP as well as other food allergies have good tolerance to HRPF.

In spite of the doubts raised by one publication (18) regarding the nutritional adequacy of hydrolysed rice protein formulas, growth was shown to be correct in these studies and also in other studies carried out using a HRPF for infants diagnosed with CMP allergy (19, 20).

Furthermore, the nutritional adequacy of such a new infant formula was shown in healthy infants who demonstrated normal growth parameters when fed a partially hydrolysed rice protein formula in a double blind, randomized trial (21).

Based on these previous trials, the main objective of the study was to evaluate the allergenicity of the HRPF and its efficacy in feeding infants diagnosed with IgE-mediated cow’s milk allergy in comparison with an extensively hydrolysed formula (40% casein and 60%whey).

Secondary objectives were to compare development of clinical tolerance to CMP and evaluate growth parameters (weight and length) of the new HRPF with that of a commercialized extensively hydrolysed formula (40% casein and 60%whey) administered to infants diagnosed with IgE-mediated cow’s milk allergy.

## Material and methods

### Study design and subjects

This was a randomized, open, parallel study of infants to compare a commercial extensively hydrolysed infant formula (EHF) with an experimental HRPF. Between September 2004 and June 2006, 92 healthy term infants were recruited into the study. Inclusion criteria were appearance of immediate reactions, mainly urticaria, erythema and angio-oedema related to the consumption of CMP (at least 2 h previously), the presence of specific IgE to cow’s milk or CMP (by skin prick test or serum antibodies) and, unless indicated otherwise, a food challenge test positive to CMP as per the usual protocol of the Hospital Department (22, 23). The study was approved by the Ethics Committee of the La Paz University Hospital (Madrid, Spain), and written informed consent was obtained from each infant’s parents.

### Study formulas

The commercialized EHF, based on extensively hydrolysed casein (40%) and whey proteins (60%) derived from cow’s milk, contained 89% of the peptides with a molecular weight of less than 1000 Daltons (EHF). The experimental formula contained partially hydrolysed rice protein supplemented with lysine and threonine to improve the nutritional quality by providing an amino acid profile similar to breast milk (HRPF).

We defined the control formula as extensively hydrolysed as it contained most (89%) of the peptides with a molecular weight of <1000 Daltons, whilst the experimental formula was defined as partially hydrolysed as it contains a greater percentage of peptides above 5000 Daltons (8% vs. 2.8%) and only 70% of the peptides with a molecular weight below 2000 Daltons. This experimental formula was more hydrolysed than those partially hydrolysed infant formulas used for prevention of CMP allergy.

The composition of the formulas complied with requirements as established by the current legislation of the European Commission Directive at the time of starting the study and is shown in Table 1. The amino acid composition of the study formulas is shown in Table 2 where it is compared to the reference values for breast milk indicated in the Directive 91/321/EEC (15).

**Table 2:** Amino acid content of the study formulas (mg/100 kcal)

Amino acids	EHF	HRPF First formula	HRPF Follow on formula	Directive 321/1991/EC
Histidine	59.7	45.8	68.4	45
Isoleucine	152.4	80.2	119.8	72
Leucine	252.6	164.2	245.3	156
Lysine	239.3	124.0	162.3	122
Methionine	54.1	45.8	68.4	29
Cystine	29.1	42.0	62.7	24
Phenylalanine	94.5	99.3	148.3	62
Tyrosine	98.1	101.2	151.2	59
Threonine	170.4	81.0	102.7	80
Valine	157.9	89.7	134.0	80
Tryptophan	36.7	30.5	42.9	30

EHF, extensively hydrolysed formula; HRPF, hydrolysed rice protein formula.

**Table 1:** Nutritional composition of the study formulas (nutrients per 100 ml infant formula)

Nutrient Unit/100 mls	EHF	HRPF first formula (0–6 months)	HRPF follow on formula (after 6 months)
Protein (g)	1.9	1.7	2.0
Lipids (g)	3.1	3.6	3.1
MCT (g)		0.7	0.6
Carbohydrates (g)	8.3	7.9	8.3
Maltodextrin (g)	8.3	6.3	6.6
Cornstarch (g)	–	1.7	1.7
Energy			
Kcal	68	71	69
KJ	287	296	290
Vitamin A (mcg)	63.0	63	65
Vitamin D (mcg)	1.1	1.1	1.7
Vitamin E (mg)	1.7	1.7	1.7
Vitamin K (mcg)	5.6	5.6	5.8
Vitamin B1 (mg)	0.7	0.7	0.8
Vitamin B2 (mg)	0.84	0.84	1.28
Vitamin B6 (mg)	1.12	1.12	1.16
Vitamin B12 (mcg)	0.3	0.3	0.3
Vitamin C (mg)	7.0	7.0	7.3
Folic acid (mcg)	5.6	5.6	15
Pantothenic Acid (mg)	0.4	0.4	0.4
Niacin (mg)	0.8	0.8	1.5
Biotin (mcg)	2.1	2.1	2.2
Choline (mg)	–	7.1	7.3
Taurine (mg)	5.0	4.9	4.8
L-carnitine (mg)	1.3	1.4	1.5
Inositol (mg)	-	3.5	3.6
Calcium (mg)	64.4	63	83
Phosphorus (mg)	36.4	35	51
Magnesium (mg)	5.6	6.3	7.3
Iron (mg)	0.8	1.1	1.1
Zinc (mg)	0.4	0.8	0.9
Copper (mcg)	42.0	42	47
Iodine (mcg)	10.0	11.2	13.1
Sodium (mg)	26.6	32	38
Potassium (mg)	60.2	63	93
Chlorine (mg)	49.0	46	78
Selenium (mcg)	0.9	1.4	1.5
Manganese (mcg)	42.0	46.2	49.6
Relation (Ca/P)	1.8	1.8	1.6
Osmolality	215 mOsm/l	200 mOsm/l	200 mOsm/l
Renal solute load	117 mOsm/l	110 mOsm/l	142 mOsm/l
Nucleotides			
Cytidine-5′-monophosphate (mg)	–	1.11	1.13
Uridine-5′-monophosphate (mg)	–	0.74	0.73
Adenosine-5′-monophosphate (mg)	–	0.38	0.38
Guanosine-5′-monophophate (mg)	–	0.22	0.22
Inosine-5′-monophosphate (mg)	–	0.22	0.22

EHF, extensively hydrolysed formula; HRPF, hydrolysed rice protein formula.

The composition of the commercialized EHF formula was the same throughout the study, whilst the new HPRF was prepared for first-age feeding (0–6 months, HRPF 1) and as a follow on formula (as of 6 months of age, HRPF 2).

Both study formulas were produced by the same company (Ordesa Group, S.L., Sant Boi de Llobregat, Barcelona, Spain). Researchers advised parents about the introduction of complementary feeding following established national guidelines (4). Feeding tolerance and adverse events were registered throughout the study period.

### Infant characteristics

Ninety-two infants diagnosed with IgE-mediated cow’s milk allergy by fulfilling the aforementioned inclusion criteria were eligible for enrolment in the study.

### Study

#### Baseline study assessments

Diagnosis was evaluated as suggested by the clinical history: typical symptoms of immediate allergy; urticaria, angio-oedema and vomiting appearing before 2 h after the intake of cow’s milk formula.

### Skin prick tests

#### Antigens for the skin test

Commercial preparations (Leti Laboratories, Barcelona, Spain) of whole cow’s milk, soya and rice as well as CMP antigens (beta-lactoglobulin, alpha-lactalbumin, casein and bovine seroalbumin) were prepared in the laboratory according to usual techniques to obtain a final solution of 10 mg protein/ml in a glycerine saline solution. Dilutions of the EHF and HRPF were prepared for skin prick testing.

#### Skin test

The skin prick was applied to the forearm and read after 15 min. Evaluation was made as per usual criteria (24), i.e. a papule of 3-mm induration compared to a negative control with saline solution. All infants exceeded this value of 3 mm.

### Biochemical measurements

Serum-specific IgE for cow’s milk and its proteins were measured, alpha-lactalbumin, beta-lactoglobulin, casein and bovine seroalbumin, using the CAP system (Pharmacia Diagnostics), with a lower cut-off point of 0.35 kU/l. The level was above 0.35 kU/l in all infants.

Furthermore, specific IgE to soy, egg white, rice and hake (but not the hydrolysed HRPF) was also measured using the CAP system also with a cut-off point of 0.35 kU/l.

### Food challenge tests for CMP

Food challenge test for CMP was carried out if not contraindicated. The reasons for contraindication of the CMP challenge test were.

1. Symptoms of generalized CMP-related anaphylaxis or oedema of the glottis.
2. Episodes of urticaria and/or general angio-oedema (not exclusively perioral), appearing within 60 mi after taking CMP, repeated two or three times, with an interval of less than 3 months since the previous episodes. These should be accompanied by positive skin prick tests of more than 3 mm and CMP-specific IgE of more than 0.35 kU/l.
3. Other situations which show specific IgE to cow’s milk or proteins of more than 3 kU/l since the probability of a positive provocation test is 90% (22)

Seventeen infants were not challenged with cow’s milk, because of anaphylaxis or anaphylactic shock, another 38 infants because of recent generalized urticaria and 33 infants who showed a value of serum IgE to CMP greater than 3 kU/l.

A food challenge test for CMP was carried out in three infants and showed positive.

### Initial formula tolerance

Initial tolerance tests to both study formulas were carried out after recruitment in all children by giving 120–150 ml of the study products.

Infants tolerant to the study products were followed up to approximately 24 months of age. At this initial stage, one infant was withdrawn from the study as a result of an allergic reaction to the EHF.

Growth parameters (weight and supine length) were also evaluated.

#### Follow-up assessments

Follow-up visits were carried out at 3 months and at 6 months after enrolment into the study or at the age of 12 months, then again at 18 and 24 months of age.

### Evaluations

At each visit, the researchers drew up a general clinical history and evaluated tolerance to the products. Written instructions were given on the introduction of complementary foods according to previously mentioned guidelines, and tolerance was checked at subsequent visits.

Growth parameters were measured, as well as determination of IgE and specific IgE levels were measured at each visit.

At the age of 12 months, total IgE levels were measured as well as specific IgE to different milk proteins and other foods (egg, fish, rice and hake). Tolerance to CMP was determined by the food challenge test, according to the established protocol, in the hospital setting with the appropriate care facilities.

### Food challenge tests

If good clinical tolerance was achieved with 100 ml of cow’s milk formula during the food challenge test, the infant was advised to follow an unrestricted diet according to age and continued tolerance was confirmed after 2 wk.

If the infant proved tolerant to CMP after the in-hospital food challenge test, and subsequent confirmation, participation in the study was discontinued.

If the infant was still allergic to CMP, follow-up was continued at 6-month intervals until approximately 24 months of age.

### Statistical analysis

Statistical analysis was carried out using the spss programme, version 11.5 (SPSS Inc., Chicago, IL, USA).

The two study groups were compared for categorical variables such as sex, type of clinical reaction, family antecedents and the presence of atopic dermatitis using the chi-square test. Student’s t-test was used for the comparative study of quantitative variables. Wilcoxon’s non-parametric test was used for any quantitative variables that did not follow normal distribution, such as the determination of serum levels of IgE and specific IgE.

Differences were considered statistically significant when p < 0.05.

## Results

Ninety-two infants were recruited (46 boys, 46 girls; mean age 4.3 months range 1.1–10.1 months). History of appearance of symptoms and clinical manifestations are shown in Tables 3 and 4 respectively. There were no statistical differences between the two groups.

**Table 3:** Baseline characteristics of the study population

Parameter	EHF	HRPF	p
Gender (M/F)	25/21	21/25	ns
Age in months	4.2 (1.5 – 8.8)	4.4 (1.1 – 10.1)	ns
Appearance first symptom			
First bottle cow’s milk formula (CMF)	23	23	ns
After 1 wk of CMF	18	19	ns
Between 1 and 3 wk of CMF	5	4	ns
Quantity of cow’s milk formula causing reaction			
Hardly any	2	0	ns
Less than 30 (ml)	17	18	ns
Between 30 and 90 (ml)	22	15	ns
More than 90 (ml)	5	13	ns
Repeated episodes	24	20	ns

EHF, extensively hydrolysed formula; HRPF, hydrolysed rice protein formula.

**Table 4:** Clinical symptoms at enrolment in the study.

Symptom	N°
Generalised urticaria	23
Urticaria of face and neck	20
Vomiting	11
Anaphylaxis	15
Anaphylactic shock	4
Perioral erythema	8
Others	11

Eighty-one infants successfully completed the study according to the protocol, after confirming clinical tolerance to the products at the baseline visit. Five infants in the experimental HRPF group abandoned follow-up for the following reasons, two refused to take the study formula, two for non-attendance at follow-up visits and one as a result of severe constipation, which was already present at the start of the study. Six infants abandoned the study in the EHF group for the following reasons, two refused to take the study formula, two for non-attendance at follow-up visits and one moved to live in another country. Thus, there were 41 infants in the HRPF group and 40 in the EHF group at the end of the study.

With regard to the primary outcome of evaluation of the clinical tolerance of the new HRPF, this was found to be similar when compared to the standard EHF, as in this EHF group, one infant showed immediate reaction of urticaria on face and trunk. However, the difference in the clinical tolerance between the groups was not statistically significant.

All infants showed negative results to the initial skin prick test to rice extract and HRPF. Specific determination of IgE to rice also proved negative.

**Fig. 1:**
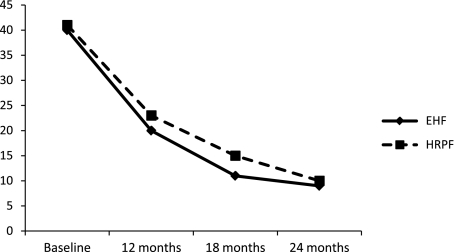
Number of infants allergic to CMP over time. EHF, Extensively hydrolysed formula; HRPF, Hydrolysed rice protein formula. p = Not significant (NS).

There was no statistically significant difference between the number of infants in the two groups who did not become tolerant to CMP during the course of the study (Fig. 1). In the EHF group, 21 infants were tolerant to CMP at 12 months, 28 at 18 months and 31 at 24 months. In the HRPF, 18 were tolerant to CMP at 12 months, 26 at 18 months and 31 at 24 months.

**Fig. 2:**
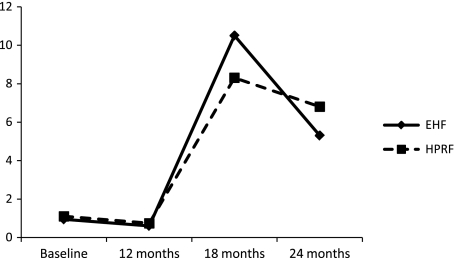
Cow’s milk protein (CMP)-Specific IgE (who did not become tolerant to CMP). EHF, Extensively hydrolysed formula; HRPF, Hydrolysed rice protein formula. p = Not significant (NS).

The specific IgE levels of those children who remained allergic to CMP at 2 yr of age are shown in Fig. 2. No statistical significant differences were found between the two groups.

As mentioned earlier, one infant in the experimental group abandoned the study because of constipation which was already been present at recruitment. All other infants showed good digestive tolerance to the study formulas (absence of vomiting and other digestive symptoms).

**Fig. 3:**
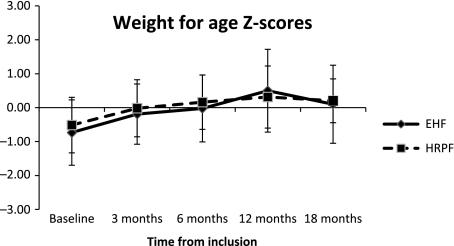
Weight for age z-scores. EHF, Extensively hydrolysed formula. HRPF, hydrolysed rice protein formula. p = not significant (NS).

**Fig. 4:**
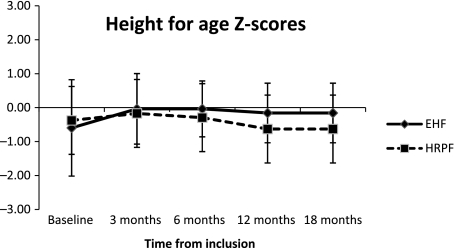
Height for age z-scores. EHF, Extensively hydrolysed formula; HRPF, hydrolysed rice protein formula. p = not significant (NS).

**Fig. 5:**
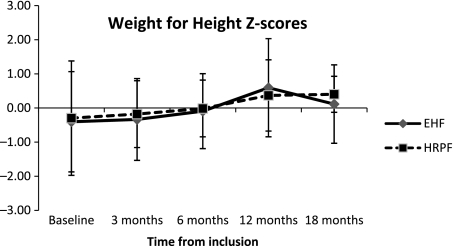
Weight for height z-scores. EHF, Extensively hydrolysed formula; HRPF, hydrolysed rice protein formula. p = not significant (NS).

Growth parameters were evaluated as z scores according to the WHO Child Growth Standards (25) and are shown in Figs 3, 4 and 5. There were no statistical differences between the groups.

## Discussion

The main objective of the study was to compare the clinical tolerance of the HRPF and its efficacy in feeding infants diagnosed with CMP allergy with an EHF. One infant in the EHF group was clinically intolerant to the product as shown by an immediate reaction. However, this is within the acceptable limit that 90% of cow’s milk allergic patients will not react to the product with 95% confidence (1–4, 6). No infant demonstrated allergenicity to the experimental formula. The results of the tests for initial allergenicity were not statistically different between the two groups studied.

The number of infants still allergic to CMP during the course of the study was similar in both groups, confirming the adequacy of the new formula when compared to the commercial EHF.

The specific IgE in both groups was markedly raised by 18 months. This may be because of the fact that those who became tolerant to CMP at 12 months of age were excluded from further follow-up.

Previous studies have indicated the low allergenicity of a HRPF. In an animal model, 7-to 12-day old guinea pigs were fed a hydrolysed rice formula, a conventional cow’s milk formula or water. A challenge was given after a sensitization period (26). When challenged with the same proteins, the group receiving the cow’s milk formula showed significantly more reactions than the group receiving the hydrolysed HRPF. In addition, when this group was challenged with the HRPF, there was no reaction and only 2 (4.4%) showed mild reactions after challenge with rice protein.

Studies in infants allergic to CMP fed with hydrolysed HRPF have shown good tolerance. In a small study, 18 infants allergic both to CMP and soy received a hydrolysed rice formula. Skin prick tests showed positive in eight children for rice and two for rice hydrolysate. Positive serology for rice using the CAP system technology was found in seven infants. DBPCFC (double-blind placebo-controlled food challenge) with hydrolysed rice formula was negative (16) indicating clinical tolerance and therefore suggesting rice hydrolysate may be used as a protein source in children with multiple food-induced reactions.

A more recent study by the same group assessed tolerance to a HRPF in children allergic to CMP (17). Allergy work-up involved skin prick tests with whole milk, α-lactalbumin, β-lactoglobulin and total caseins as well as specific IgE determinations (CAP technology) to whole milk. Sensitization to rice and rice hydrolysate formula was measured. The specific IgE was evaluated together with immunoblotting to CMP, to rice and hydrolysed rice formula. Positive IgE levels were found in 21/91 infants for rice and 4/91 hydrolysed rice infant formula. Immunoblotting was positive to rice proteins for 70/96 infants. Response was weakly positive to hydrolysed rice infant formula in 6/96. The DBPCFC was negative to hydrolysed rice protein formula in all cases. This suggests that hydrolysis reduces antigenicity of rice protein by abolishing IgE-binding capacity.

These studies are relevant to our study as they provide further data on the clinical tolerance to hydrolysed rice protein formulas in children with immediate reactions to CMP.

In this study, the HRPF was generally well tolerated, with the exception of two infants who refused the product after receiving another product over a weekend. One of the main complaints given by parents is that infants reject hydrolysed formulas because of an unpleasant taste. A recent double-blind study to evaluate the palatability of different formulas used to feed infants with CMP allergy showed that the soy formulas and HRPF had the best taste scores followed by different hydrolysed formulas (27). Good oral tolerance because of its pleasant odour, taste and flavour was found with the HRPF in healthy infants (21).

The good palatability of the new formula offers an alternative in the older infant used to breast milk feeding when there is a need to switch to a hypoallergenic formula.

Other further studies have documented the good tolerance of the hydrolysed rice protein formula (16, 17, 19, 28).

The capacity for a partially hydrolysed rice protein formula to sustain normal growth was shown in healthy infants when consumed for the first 4 months after birth (21). In this study, comparison was made with a group of infants receiving a standard commercial formula.

Two studies evaluated the nutritional value of a rice hydrolysate in infants diagnosed with CMP. They showed similar growth to those receiving a soy formula in early life (19) and during the weaning period of 6–12 months (20). This was contradicted in a study by Savino et al. (18) who found that growth was impaired in children with atopic dermatitis and CMP who received a rice hydrolysate formula compared to another formula. In these studies quoted, the same hydrolysed rice protein formula was administered to the allergic infants.

In this study, growth of the infants receiving the HRPF was within normal parameters for both weight and height when compared to the WHO Growth Standards. There was no statistical difference between the groups in growth.

No biochemical markers of plasma amino acid levels were measured during the study.

One final point for consideration is the recent concern, raised by the Food Standards Agency of the United Kingdom, regarding the question of arsenic levels in rice drinks, especially the content of inorganic arsenic that is considered as more harmful (29). There are no EU regulations on the contents of arsenic in food.

In the United Kingdom, there is a general limit of 1 mg/kg (or 1 ppm) for arsenic in food.

The study carried out by the Food Safety Authority (FSA) on the arsenic content of rice drinks found an average concentration of 0.023 mg/kg (0.023 ppm) of total arsenic and 0.012 mg/kg (0.012 ppm) of inorganic arsenic.

The arsenic content of the hydrolysed rice infant formula administered in this study has been analysed. The total arsenic content of the reconstituted product is 6.4 times less than the values quoted in the FSA study, and the inorganic arsenic is more than six times lower than the values found in the FSA study.

The European Food Safety Authority (EFSA) has recently published a Scientific Opinion on Arsenic in Food (30). The recommendation of the Panel was that so as to refine risk assessment of inorganic arsenic, there is a need to produce speciation data for different food commodities to support dietary exposure and dose–response data for the possible health effects.

In this EFSA report, the total arsenic content of rice-based infant food was found to be 0.158 mg/kg of dry product. The hydrolysed rice protein formula in our study contained six times less than this value.

Furthermore, EFSA assumed that 70% of total arsenic is inorganic so that the concentration of inorganic arsenic in rice-based infant food was 0.110 mg/kg. The inorganic arsenic content of the study formula contains 7.3 times less than this value.

In summary, in this study, the HRPF was well tolerated by infants with moderate to severe symptoms of IgE-mediated CMP allergy. Children receiving this formula showed similar growth and development of clinical tolerance to those receiving an EHF. In accordance with current guidelines, this HRPF was tolerated by more than 90% of children with CMP allergy and therefore could provide an adequate and safe alternative to CMP-hydrolysed formulas for these infants**.**
